# Prevalence evolution of SARS-CoV-2 infection in the city of São Paulo, 2020–2021

**DOI:** 10.11606/s1518-8787.2021055003970

**Published:** 2021-10-05

**Authors:** José Olimpio Moura de Albuquerque, Gabriela Akemi Kamioka, Geraldine Madalosso, Selma Anequini Costa, Paula Bisordi Ferreira, Francisco Alberto Pino, Ana Paula Sayuri Sato, Ana Carolina Aguiar de Carvalho, Ana Beatriz Pagliaro Amorim, Caroline Cotrim Aires, Ana Paula Arruda Geraldes Kataoka, Elisa San Martin Mouriz Savani, Thirsa Alvares Franco Bessa, Breno Souza de Aguiar, Marcelo Antunes Failla, Edson Aparecido dos Santos, Edjane Maria Torreão Brito, Maria Cristina Honório dos Santos, Solange Maria Saboia e Silva, Luiz Artur Vieira Caldeira, Luiz Carlos Zamarco, Sandra Maria Sabino Fonseca, Marcia Maria de Cerqueira Lima, Ivanilda Argenau Marques, Fabiana Érica Vilanova da Silva, Paula Regina Glasser, Patrícia Carla Piragibe Ramos Burihan, Cinthya Luzia Cavazzana, Debora Silva de Mello, Alessandra Cristina Guedes Pellini, Fernando Yoshiki Nishio, Fernanda Miyashiro Kian, Elza de Santana Braga, Nilza Maria Piassi Bertelli, Wagner Fracini, Marcelo Dell Áquila Gonçalves, Paulete Secco Zular, Regiane de Santana Piva, Eduardo Masi

**Affiliations:** I Secretaria Municipal de Saúde Coordenadoria de Vigilância em Saúde São PauloSP Brasil Secretaria Municipal de Saúde. Coordenadoria de Vigilância em Saúde.São Paulo, SP, Brasil; II Universidade de São Paulo Faculdade de Saúde Pública São PauloSP Brasil Universidade de São Paulo. Faculdade de Saúde Pública. São Paulo, SP, Brasil; III Secretaria de Agricultura e Abastecimento Instituto de Economia Agrícola São PauloSP Brasil Secretaria de Agricultura e Abastecimento. Instituto de Economia Agrícola. São Paulo, SP, Brasil; IV Secretaria Municipal de Saúde São PauloSP Brasil Secretaria Municipal de Saúde.São Paulo, SP, Brasil; V Universidade Nove de Julho Faculdade de Medicina São PauloSP Brasil Universidade Nove de Julho. Faculdade de Medicina. São Paulo, SP, Brasil; VI Secretaria Municipal de Saúde Coordenadoria Regional de Saúde São PauloSP Brasil Secretaria Municipal de Saúde. Coordenadoria Regional de Saúde. São Paulo, SP, Brasil

**Keywords:** Adult, COVID-19, epidemiology, COVID-19 Serological Testing, Health Surveys

## Abstract

**OBJECTIVE:**

To estimate the evolution of the prevalence of SARS-CoV-2 virus infection among residents aged 18 years or over in the municipality of São Paulo.

**METHODS:**

This is a population-based household survey conducted every 15 days, between June and September 2020, and January and February 2021. In total, the study comprised 11 phases. The presence of antibodies against SARS-CoV-2 was identified in venous blood using a lateral flow test, Wondfo Biotech. In the last phase, the researchers combined it with an immunoenzymatic test, Euroimmun. The participants also answered a semi-structured questionnaire on sociodemographic and economic factors, and on social distancing measures. Prevalence estimates and the 95% confidence interval were estimated according to regions, Human Development Index, sex, age group, ethnicity, education, income, and variables associated with risk or prevention of infection. To compare the frequencies among the categories of each variable, the chi-square test with Rao-Scott correction was used, considering a significance level of 5%.

**RESULTS:**

In total, 23,397 individuals were interviewed and had their samples collected. The estimated prevalence of antibodies against SARS-CoV-2 ranged from 9.7% (95%CI: 7.9–11.8%) to 25.0% (95%CI: 21.7–28.7). The prevalence of individuals with antibodies against the virus was higher among black and brown people, people with lower schooling and income, and among residents of regions with lower Human Development Index. The lowest prevalences were associated with recommended measures of disease protection. The proportion of asymptomatic infection was 45.1%.

**CONCLUSION:**

The estimated prevalence of the infection was lower than the cumulative incidence variation, except for the last phase of the study. The differences in prevalence estimates observed among subpopulations showed social inequality as a risk of infection. The lower prevalence observed among those who could follow prevention measures reinforce the need to maintain social distancing measures as a way to prevent SARS-CoV-2 infection.

## INTRODUCTION

In December 2019, the World Health Organization (WHO) received a notification of pneumonia outbreak in Wuhan, Hubei Province, People’s Republic of China. The etiological agent was quickly identified: a new coronavirus called SARS-CoV-2. On January 30, 2020, WHO declared the disease outbreak caused by the virus (COVID-19) a Public Health Emergency of International Concern (PHEIC), the highest WHO alert level according to International Health Regulations (IHR). On March 11, 2020, WHO declared the outbreak of COVID-19 a global pandemic^1^.

In Brazil, the Ministry of Health (MoH) declared the outbreak a Public Health Emergency of National Concern (PHENC) on February 3, 2020^2^. The first case was diagnosed on February 26, 2020, and on March 20, 2020, MoH announced the community transmission of COVID-19 in the national territory^3^.

From the date of the first case until March 28, 2021, the virus infected 12,490,362 people and caused 310,550 deaths, with an incidence rate of 5,943.6 per 100,000 inhabitants, and a mortality rate of 147.8 per 100,000 inhabitants^4^.

In the municipality of São Paulo (MSP), until March 26, 2021, the health report system (e-SUS Notifica) had 2,390,256 cases of acute respiratory infection (ARI), of which 609,380 (25.5%) were confirmed for SARS-CoV-2 infection. In the Epidemiological Surveillance Information System of ARI (SIVEP Gripe), 161,758 cases of severe acute respiratory syndrome (SARS) in the city were reported, of which 90,049 (55.7%) were confirmed for COVID-19. Among these cases, 21,051 (3%) resulted in death.

Given the epidemiological situation of COVID-19 in the MSP, on March 23, 2020, the city council adopted strategies for reducing the disease transmission, establishing voluntary quarantine with the closure of non-essential businesses^5^. On May 29, 2020, Decree No. 59,473 gradually reopened some non-essential services^6^; therefore, it became extremely important to know the serological situation of the population regarding COVID-19 infection to support decision-making.

The researchers designed this serial serological survey to represent people with 18 years or older in the MSP and used population-based data to direct strategies to combat the pandemic and evaluate the effects of COVID-19 actions to prevent and control the disease. In this sense, our study aimed to estimate the prevalence of SARS-CoV-2 virus infection in these adults living in the city, the proportion of asymptomatic individuals with positive tests, and describe the evolution of the infection prevalence.

## METHODS

This is a serial serological survey to estimate the prevalence of SARS-CoV-2 infection in the city of São Paulo.

### Study Area

The capital of the state of São Paulo has a Human Development Index (HDI) of 0.805 and an estimated population in 2020 of 11.9 million inhabitants, of which 9.2 million were 18 years or older. The city also has a large economic and social disparity, mainly in the education level, income, and housing – the Gini Index in 2010, for instance, was 0.6453^7^.

**Figure 1:**
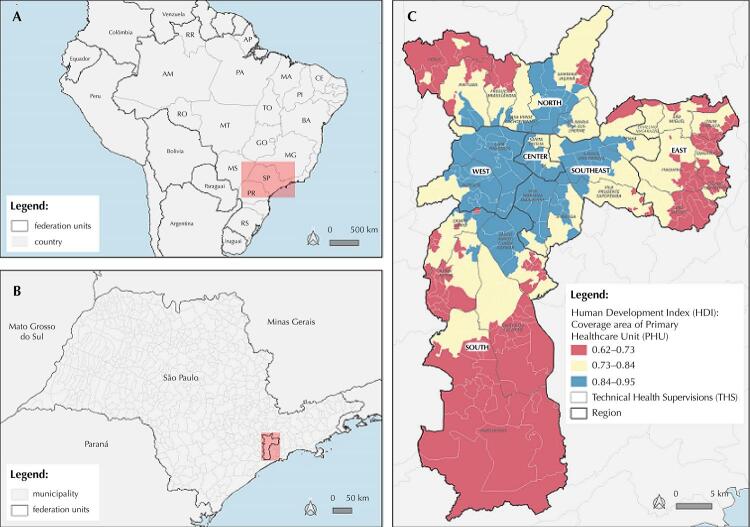
Distribution of Human Development Index (HDI) by Coverage Area of Primary Healthcare Unit (CA-PHU) and region. Municipality of São Paulo, 2020.

For planning healthcare actions, the city was divided into six regions – North, Center, West, Southeast, East and South –, 27 Technical Health Supervisions, 472 primary healthcare units (PHU), and their respective coverage areas (CA) (Figure 1).

Figure 1 shows the social and economic disparities between and within the regions of the MSP.

### Sample Methodology

In 2020, we conducted a pilot study in June and seven cross-sectional studies periodically in different samples, every 15 days, from June to September. In 2021, four other studies were carried out in January and February. In each phase, a stratified probabilistic sample with simple casual sampling was used within each stratum^8^.

For a prevalence ranging from 5% to 20%, we determined the sample size so that the estimates had coefficients of variation lower than 15% for the MSP, and lower than 30% for the regions, except for the Central and West regions, which have a smaller number of PHU. This process resulted in eight dwellings per stratum. The researchers increased the sample size to 12 dwellings to maintain the accuracy of the estimates affected by non-response (closed households or refusals to participate in the study). In the Central and West regions, the sample size per CA-PHU was also increased to 15 to compensate the smaller number of PHU in the region.

Statistically, this is a single stage sampling (the dwelling is the sample unit); however, operationally, the selection comprised two stages: in the second stage, the resident was selected in the household using the last birthday method. The selected person collected material for laboratory analysis and was interviewed. If the selected property was closed or the resident was not at home at the time of the visit, the PHU’s team would return twice.

### Database

The addresses were accessed from a database composed by three different types of records updated between the study phases: a) residential property taxpayers (IPTU) of 2020; b) hydrometers of sanitation company (SABESP) of 2017; c) Family Health Strategy (FHS). The distribution of dwellings by record was unequal in the city. Some areas did not have representativeness from one of the records or the number was insufficient in the sample. The number of dwellings in each stratum was selected from the database, proportionally to the number of dwellings in each record.

### Testing and Questionnaire

The researchers used SARS-CoV-2 Antibody test^®^ (Wondfo Biotech, Guangzhou, China), which detects IgM/IgG antibodies against the virus, without discriminating the type of immunoglobulin. In Brazil, the test is distributed by the MoH under the name “One Step COVID-2019 Test^®^”, and the legal manufacturer is Celer Biotecnologia S/A^9^.

The test is based on the principle of lateral flow immunochromatographic for the detection of IgG/IgM antibodies against SARS-CoV-2 in human blood, serum or plasma^10^.

In this survey, we used venous blood samples to obtain the serum, since the validation study of the OneStep Wondfo Test indicated an increase of sensitivity with serum sample in comparison with the capillary blood obtained by digital puncture. Pellanda et al.^11^ estimated a sensitivity of 84.8% and a specificity of 99.0% by assessing the results of four validated studies.

In the last phase, to increase the sensitivity and the specificity of virus detection, the samples were also processed using an immunoenzymatic test (anti-SARS-CoV-2 ELISA) from Euroimmun, which uses the S1 spike protein as an antigen for the detection of IgG antibodies against the virus in the serum^12,13^.

The official laboratory results were reported to the study participants. They answered a semi-structured questionnaire with questions about sex, age, schooling, ethnicity (self-reported), family income, household size, symptoms potentially related to COVID-19, healthcare service use, previous SARS-CoV-2 test, contact with suspected or confirmed cases of COVID-19. Also, the interviewees were questioned about their work regime, social distancing measures adopted, facemask use, visit to non-essential places and public transportation use.

### Data Analysis

Data were included in a standardized electronic form in FormSUS/DATASUS, version 3.0^14^. Data processing and analysis were performed using the statistical packages R and STATA version 13. Indeterminate results were classified in the analysis as negative.

Data were analyzed considering five regions of the city: Central-West; East; North; Southeast; and South. Prevalence estimates were weighted according to the sampling design.

Prevalence and their 95% confidence intervals (CI) were estimated according to the region and HDI of the city, sex, age, ethnicity, education, income and presence of symptoms, risk factors, recommended measures for prevention and control of the disease and social distancing. Moreover, we calculated the proportion of asymptomatic infections.

For statistical analyses, four phases were randomly chosen from the 11 phases conducted in this study. The Rao-Scott chi-square test was used to compare the frequencies between the categories of each variable, considering a significance level of 5%.

### Ethical Aspects

The study was approved by the Brazilian’s National Ethics Committee (CAAE 32947920.3.0000.0008). Blood samples were collected and the individuals were interviewed only after signing an informed consent form.

## RESULTS

Out of 63,372 selected individuals with 18 years or over, residing in the five regions, 23,397 (36.9% participation rate) were interviewed and collected samples.

Table 1 shows the prevalence estimates of antibodies against SARS-CoV-2 in the city and their respective 95%CI for each phase of the study. All values found were within the range of CI variation of the previous phases, except in the last phase, when the researchers used two laboratory tests to increase the sensitivity and the specificity of virus detection.

**Table 1:** Sample size, number of sample collections, positive results and prevalence estimates of SARS-CoV-2 infection and respective 95% confidence intervals by study phase. Municipality of São Paulo, 2021.

Study phase	Period of sample collection	Sample size	Number of sample collections	Number of positive results (SARS-CoV-2)	Prevalence estimate (95%CI)
Pilot	06/10/2020 to 06/17/2020	5,664	2,645	247	9.5 (7.9–11.4)
1	06/29/2020 to 07/02/2020	5,772	2,481	261	9.7 (7.9–11.8)
2	07/13/2020 to 07/16/2020	5,760	2,323	282	11.1 (9.6–12.9)
3	07/28/2020 to 07/30/2020	5,760	2,529	296	10.9 (9.2–12.9)
4	08/11/2020 to 08/13/2020	5,760	2,447	298	11.0 (9.0–13.3)
5	08/25/2020 to 08/27/2020	5,760	2,225	303	13.9 (11.8–16.2)
6	09/08/2020 to 09/10/2020	5,760	2,125	270	11.9 (10.0–14.1)
7	09/22/2020 to 09/24/2020	5,760	2,012	244	13.6 (11.6–15.8)
8	01/05/2021 to 01/07/2021	5,760	1,908	278	14.1 (11.7–16.8)
9	01/19/2021 to 01/21/2021	5,760	1,795	270	13.9 (11.7–16.4)
10	02/02/2021 to 02/04/2021	5,760	1,742	281	16.0 (13.1–19.3)
11	02/16/2021 to 02/18/2021	5,760	1,780	438	25.0 (21.7–28.7)

Table 2 shows the prevalence estimates of SARS-CoV-2 infection in phases 1, 4, 7, and 11. The results of phase 11 with only the rapid test (11a) and with the addition of the ELISA test (11b) are presented separately.

**Table 2:** Prevalence estimates of SARS-CoV-2 infection by demographic and clinical characteristics and study phases (1, 4, 7 and 11). Municipality of São Paulo, 2021.

Variables	Phase 1			Phase 4			Phase 7			Phase 11 (a*)			Phase 11 (b*)		
				
	n (%)	Prevalence (95%CI)	p	n (%)	Prevalence (95%CI)	p	n (%)	Prevalence (95%CI)	p	n (%)	Prevalence (95%CI)	p	n (%)	Prevalence (95%CI)	p
Region			0.892			0.027			< 0.001			0.027			0.487
Center	225 (14.2)	10.1 (3.7–24.8)		225 (6.3)	5.2 (2.7–9.6)		167 (4.8)	5.5 (2.3–12.5)		140 (7.8)	9.5 (6.0–14.7)		140 (9.2)	19.4 (7.9–40.3)	
East	697 (18.6)	10.0 (7.9–12.5)		636 (19.3)	12.3 (9.5–15.8)		568 (16.4)	11.7 (8.4–16.1)		472 (24.1)	17.8 (13.9–22.5)		472 (20.5)	26.0 (21.1–31.6)	
North	430 (14.4)	8.5 (5.7–12.5)		463 (12.3)	8.3 (6.0–11.5)		372 (16.7)	13.8 (9.7–19.1)		327 (21.6)	19.0 (14.4–24.7)		327 (17.2)	26.0 (20.7–32.1)	
Southeast	564 (18.5)	8.4 (5.8–12.1)		501 (18.9)	10.6 (7.5–14.8)		399 (15.9)	10.3 (6.7–15.4)		363 (12.8)	9.4 (6.0–14.2)		363 (16.7)	20.1 (16.2–26.6)	
South	565 (34.3)	10.7 (7.8–14.6)		623 (43.2)	14.1 (9.5–20.4)		510 (46.3)	19.8 (15.7–24.8)		491 (33.7)	15.4 (10.6–22.0)		491 (36.4)	28.6 (21.7–36.7)	
Demographic															
Age			0.145			0.241			0.011			0.347			0.174
18 to 34 years	622 (28.8)	10.0 (7.3–13.5)		672 (39.6)	13.1 (9.5–17.9)		540 (21.7)	10.8 (7.3–15.7)		411 (31.1)	16.8 (12.6–22.2)		411 (31.6)	29.4 (23.9–35.6)	
35 to 49 years	753 (32.4)	9.6 (5.9–15.3)		680 (28.7)	12.0 (8.1–17.6)		547 (35.4)	19.1 (14.8–24.4)		489 (23.5)	13.7 (9.0–20.3)		489 (21.3)	21.3 (16.0–27.7)	
50 to 64 years	617 (30.0)	12.4 (8.9–17.1)		597 (17.8)	8.1 (5.5–11.6)		518 (29.5)	14.4 (11.0–18.5)		491 (21.8)	11.6 (8.2–16.2)		491 (29.3)	27.0 (19.5–36.0)	
65 or more years	489 (8.8)	5.1 (2.7–9.5)		499 (13.9)	9.3 (6.1–14.0)		441 (13.4)	9.2 (5.9–14.3)		402 (23.7)	16.6 (12.8–21.2)		402 (17.8)	21.4 (17.0–26.5)	
Sex			0.397			0.264			0.973			0.053			0.034
Men	892 (33.8)	8.6 (6.6–11.1)		881 (33.8)	9.4 (6.5–13.4)		703 (39.2)	13.5 (10.4–17.4)		1,184 (28.7)	11.9 (9.3–15.0)		1,184 (29.7)	20.7 (17.1–24.8)	
Women	1,583 (66.2)	10.1 (7.6–13.3)		1,567 (66.2)	12.0 (9.5–15.1)		1,313 (60.8)	13.6 (11.2–16.5)		609 (71.3)	16.0 (13.1–19.5)		609 (70.3)	27.3 (22.7–32.6)	
Unknown	6 (-)	-		0 (-)	-		0 (-)	-		0 (-)	-		0 (-)	-	
Race/color			0.029			0.002			< 0.001			0.005			0.018
White	1,292 (40.7)	7.3 (5.5–9.5)		1,263 (36.3)	7.5 (5.7–9.9)		1,002 (37.4)	9.5 (7.2–12.4)		827 (39.9)	11.6 (9.5–14.1)		827 (43.2)	21.6 (16.7–27.4)	
Black/Brown	1,137 (58.0)	12.7 (9.5–16.9)		1,128 (62.0)	15.1 (11.6–19.6)		969 (61.4)	18.8 (15.5–22.7)		924 (59.7)	18.0 (14.2–22.6)		924 (56.4)	29.3 (24.8–34.2)	
Asian	42 (1.3)	7.1 (1.2–33.1)		41 (1.8)	13.7 (2.9–46.1)		23 (1.3)	15.1 (4.2–41.8)		23 (0.4)	3.8 (0.0–29.4)		23 (0.4)	6.2 (1.1–28.0)	
Indigenous	4 (0.0)	-		4 (0.0)	-		1 (0.0)	-		1 (0.0)	-		1 (0.0)	-	
Unknown	6 (-)	-		12 (-)	-		21(-)	-		18 (-)	-		18 (-)	-	
Education level			< 0.001			0.003			< 0.001			0.186			0.094
Middle School	764 (39.7)	14.9 (10.4–20.8)		730 (33.6)	15.6 (11.4–21.1)		661 (37.2)	17.8 (14.3–22.0)		565 (32.1)	15.9 (10.7–23.0)		565 (34.4)	29.1 (21.0–38.9)	
High school	1,007 (35.1)	8.6 (6.7–10.8)		963 (46.8)	11.9 (8.6–16.2)		803 (48.0)	16.0 (12.5–20.1)		684 (40.7)	15.7 (12.4–19.7)		684 (39.3)	26.0 (21.7–30.7)	
Higher education	609 (16.3)	4.9 (3.1–7.4)		580 (16.1)	5.9 (3.9–8.9)		409 (11.5)	6.0 (3.6–9.7)		351 (20.3)	11.2 (8.1–15.1)		351 (20.6)	19.4 (15.2–24.4)	
Illiterate	87 (9.0)	29.5 (11.6–57.0)		122 (3.6)	9.4 (4.1–19.7)		78 (3.3)	14.7 (5.7–33.0)		94 (7.0)	24.7 (12.8–42.4)		94 (5.8)	35.5 (21.8–52.1)	
Unknown	14 (-)	-		53 (-)	-		65 (-)	-		99 (-)	-		99 (-)	-	
Clinics															
Asymptomatic			< 0.001			< 0.001			< 0.001			< 0.001			< 0.001
Yes	1,708 (43.6)	6.0 (4.0–8.8)		1,609 (40.5)	6.4 (4.5–9.1)		1,351 (35.3)	7.5 (5.6–10.1)		1,111 (45.7)	11.3 (8.3–15.1)		1,111 (45.1)	19.0 (14.9–23.8)	
No	766 (56.4)	18.3 (14.7–22.6)		838 (59.5)	20.7 (16.4–25.8)		661 (64.7)	24.6 (20.7–28.9)		601 (54.3)	21.1 (17.4–25.5)		601 (54.9)	36.4 (30.6–42.6)	
Unknown	7 (-)	-		1 (-)	-		4 (-)	-		81 (-)	-		81 (-)	-	

(a)* = rapid test; (b)* = rapid test + ELISA.

**Figure 2:**
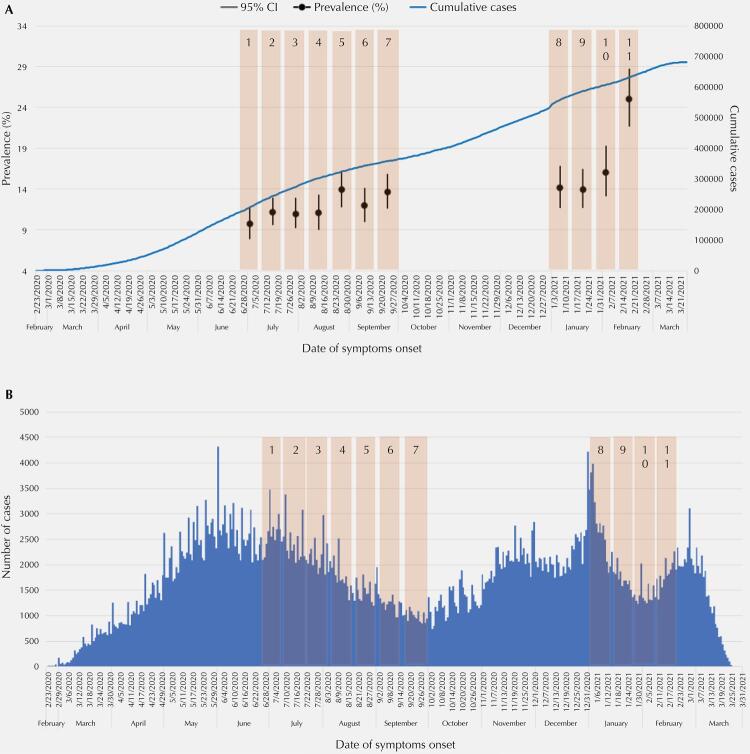
(A) Prevalence estimates and 95% confidence interval of SARS-CoV-2 infection by study phases; cumulative cases of SARS-CoV-2 infection by date of symptoms onset; (B) Distribution of COVID-19 cases by date of symptoms onset and study phases. Municipality of São Paulo, 2021.

The prevalence estimates of this study did not follow the evolution of the accumulated cases of SARS-CoV-2 infection in the MSP, except for the last phase, as shown in Figure 2A.

The design effect (deff) varied between the study phases, from 1.93 in phase 7 to 3.09 in the last phase; in phase 1, the deff was 2.71, and in phase 4, 2.95.

The coefficients of variation of prevalence estimates for the municipality were below 15% in all phases and below 30% in all regions, except for the Central-West region.

There was a significant difference between the regions in phases 4 and 7. The Central-West region had the lowest estimates, whereas the Southern region had the highest.

Table 2 also presents the prevalence estimates of SARS-CoV-2 infection, according to demographic and clinical characteristics. Regarding age, the estimated prevalences varied between the phases of the study: in phase 1, the highest prevalence was found in the age group from 50 to 64 years; in phase 4, from 18 to 34 years; in phase 7, from 35 to 49 years, and in phase 11, from 18 to 34 years. Only in phase 7, the difference among age groups was significant.

In all phases, women had the highest prevalence, especially in phase 11, when the difference was statistically significant.The estimated prevalence of black and brown people was the highest in all phases. There was a significant difference in ethnicity in all phases.

The highest prevalence estimates were found in the groups with the lowest education levels. Table 2 shows significant differences between education levels in phases 1, 4, and 7.

The proportion of asymptomatic individuals who tested positive ranged from 35.3% (95%CI: 29.5–41.6) in phase 7 to 45.1% (95%CI: 38.2–52.2) in phase 11. In phase 1, the proportion was 43.6% (95%CI: 37.4–50.0) and in phase 4, 40.5% (95%CI: 31.8–49.9). As shown in Table 2, the prevalence of cases without symptoms increased throughout the study phases.

Table 3 shows the prevalence estimates of SARS-CoV-2 infection according to socioeconomic factors and recommended measures adopted, for phases 1, 4, 7, and 11.

**Table 3:** Prevalence estimates of SARS-CoV-2 infection by socioeconomic factors, recommended measures and study phases (1. 4. 7 and 11). Municipality of São Paulo, 2021.

Variables	Phase 1			Phase 4			Phase 7			Phase 11 (a*)			Phase 11 (b*)		
				
	n (%)	Prevalence (95%CI)	p	n (%)	Prevalence (95%CI)	p	n (%)	Prevalence (95%CI)	p	n (%)	Prevalence (95%CI)	p	n (%)	Prevalence (95%CI)	p
Economic															
HDI			0.015			0.021			< 0.001			0.068			0.281
Range A (0.84 to 0.95)	390 (22.6)	8.3 (4.2–15.7)		347 (14.8)	6.2 (3.8–10.0)		270 (8.7)	4.6 (2.7–8.0)		204 (16.8)	10.7 (7.1–15.7)		204 (18.5)	20.1 (12.3–30.9)	
Range B (0.73 to 0.84)	1,401 (39.6)	7.5 (5.7–9.9)		1,304 (54.7)	12.1 (9.3–15.6)		1,038 (54.8)	15.3 (12.1–19.1)		978 (50.1)	14.4 (11.2–18.3)		978 (51.5)	25.4 (20.8–30.6)	
Range C (0.62 to 0.73)	690 (37.8)	16.2 (12.9–20.1)		797 (30.5)	14.0 (9.7–19.8)		708 (36.4)	19.3 (15.7–23.4)		611 (33.1)	18.4 (14.3–23.4)		611 (30.1)	28.8 (24.0–34.1)	
Income			0.040			< 0.001			0.037			0.054			0.005
Class A/B	132 (2.1)	2.3 (0.6–8.7)		144 (3.3)	4.4 (1.7–10.8)		77 (1.8)	8.9 (1.3–42.5)		64 (1.8)	3.9 (0.9–15.2)		64 (1.2)	4.7 (1.3–15.0)	
Class C	1,182 (47.5)	8.9 (6.2–12.7)		1,013 (34.2)	8.3 (6.5–10.7)		842 (35.8)	10.2 (7.9–13.1)		763 (45.6)	12.7 (9.9–16.0)		763 (48.3)	23.7 (18.8–29.4)	
Class D/E	974 (41.3)	12.5 (9.5–16.2)		1,055 (57.3)	18.2 (13.7–23.7)		913 (52.9)	18.9 (15.5–23.0)		757 (42.9)	17.9 (13.3–26.7)		757 (40.5)	29.8 (23.7–36.7)	
Not informed	193 (9.2)	10.3 (6.1–16.8)		211 (5.2)	4.7 (2.4–9.1)		160 (9.5)	11.4 (5.5–21.8)		142 (9.7)	13.0 (0.7–22.6)		142 (10.0)	23.4 (15.7–33.4)	
Unknown	21 (-)	-		25 (-)	-		24 (-)	-		67 (-)	-		67 (-)	-	
Recommended measures															
Work situation			< 0.001			0.002			0.097			0.593			0.121
Unemployed	411 (26.6)	15.4 (9.0–25.0)		427 (29.8)	18.1 (11.6–27.1)		340 (15.3)	13.8 (9.2–20.0)		291 (15.5)	14.7 (9.8–21.3)		291 (14.4)	23.4 (17.1–31.3)	
Telework	439 (8.5)	3.9 (2.6–6.0)		381 (8.0)	4.4 (2.2–8.7)		277 (10.5)	8.7 (4.8–15.0)		213 (13.5)	13.6 (8.5–21.1)		213 (11.4)	19.8 (14.1–27.2)	
Employed	591 (33.6)	14.1 (10.7–18.3)		613 (26.6)	11.9 (9.1–15.5)		535 (33.5)	18.1 (13.7–23.5)		519 (35.3)	17.2 (12.5–23.2)		519 (37.0)	31.0 (24.3–38.7)	
Not active	874 (25.1)	7.4 (4.9–11.1)		909 (31.8)	10.6 (7.5–14.7)		770 (37.6)	13.5 (10.9–16.5)		665 (32.7)	13.6 (10.5–17.4)		665 (33.8)	24.1 (18.7–30.5)	
Mixed work	134 (6.2)	10.3 (6.0–17.2)		107 (3.9)	9.0 (4.5–17.2)		81 (3.1)	7.7 (2.2–23.4)		72 (3.0)	9.8 (4.8–19.0)		72 (3.4)	19.6 (11.7–30.9)	
Unknown	32 (-)	-		11 (-)	-		13 (-)	-		33 (-)	-		33 (-)	-	
Use of public transportation			0.013			0.607			0.437			0.249			0.022
No	1,944 (75.0)	9.2 (7.0–11.8)		1,755 (74.0)	11.3 (8.9–14.1)		1,367 (66.7)	13.6 (11.4–16.2)		969 (53.5)	12.8 (10.3–15.7)		969 (50.7)	20.2 (17.2–23.6)	
Yes	446 (25.0)	14.0 (10.9–17.7)		586 (26.0)	10.3 (7.7–13.6)		575 (33.3)	15.5 (11.7–20.4)		661 (46.5)	15.8 (11.6–21.2)		661 (49.3)	28.0 (22.1–34.7)	
Unknown	91 (-)	-		107(-)	-		74 (-)	-		163 (-)	-		163 (-)	-	
Social distancing			< 0.005			0.342			0.320			0.006			0.005
Totally	1,700 (58.4)	8.0 (5.9–10.1)		1,871 (73.9)	10.3 (8.1–12.9)		1,566 (70.3)	12.7 (10.6–15.1)		1,260 (66.3)	13.0 (10.4–16.2)		1,260 (66.8)	22.6 (18.4–27.4)	
Partially	701 (37.8)	13.6 (10.7–17.1)		530 (24.3)	13.1 (8.8–19.0)		412 (27.9)	17.6 (13.2–23.1)		475 (32.9)	19.5 (15.8–23.9)		475 (32.2)	32.9 (28.1–38.1)	
Not adopted	41 (3.8)	18.9 (69–42.1)		35 (1.8)	17.9 (6.3–41.2)		24 (1.8)	20.0 (2.5–70.6)		22 (0.8)	10.0 (5.4–17.7)		22 (1.1)	23.8 (8.7–50.4)	
Unknown	39 (-)	-		12 (-)	-		14 (-)	-		36 (-)	-		36 (-)	-	
Use of mask						0.677			0.841			0.155			0.022
Always				2,108 (87.4)	11.0 (8.9–13.7)		1,734 (89.1)	13.6 (11.6–15.9)		1,496 (82.4)	13.9 (11.7–16.6)		1,496 (84.1)	24.4 (20.7–28.6)	
Mostly	Data obtained from phase 2			225 (9.7)	10.9 (6.6–17.6)		185 (7.6)	13.2 (6.9–23.7)		178 (13.8)	22.7 (14.8–33.1)		178 (11.1)	31.6 (22.6–42.2)	
Sometimes				68 (2.6)	15.3 (6.5–31.8)		53 (3.0)	21.2 (6.1–52.7)		58 (3.5)	16.9 (6.3–38.2)		58 (3.6)	29.4 (13.8–52.0)	
Never				9 (0.3)	7.5 (0.6–48.9)		7 (0.3)	–		5 (0.3)	3.7 (0.9–13.9)		5 (1.2)	85.0 (37.7–98.1)	
Unknown				17 (-)	-		27 (-)	-		41 (-)	-		41 (-)	-	

(a)* = rapid test; (b)* = rapid test + ELISA.

Prevalence estimates were inversely related to the family income category that were distributed in A/B (≥ R$ 8,641.00), C (R$ 2,005.00 to R$ 8,640.00) and D/E (≤ R$ 2,004.00). Moreover, we used HDI ranges to estimate regional differences: range A (0.84 to 0.95) had the lowest prevalence, whereas range C (0.62 to 0.73) had the highest estimates. The difference among HDI ranges and family income categories were significantly different, except in phase 11 (Table 3).

Regarding the work situation of the interviewees, the study showed that those in telework had lower prevalence when compared to the other categories, especially in phases 1 and 4 (3.9% and 4.4%, respectively). Likewise, those who did not use public transportation and adopted social distancing measures had the lowest prevalence estimates. The difference was significantly in phases 1 and 11.

The difference between the categories for mask use – “always,” “most of the time,” and “sometimes” – was significant in phase 11 (24.4%).

Interviews and sample collection could not be conducted in 39,169 (62.6%) households. In 11,362 (29%) addresses, the selected dwelling was not identified, and in 6,489 (16.6%), the property was closed. In 10,443 (26.7%) dwellings, the selected individual refused to participate; in 6,436 (16.4%), they were not present at the visit, in 4,404 (11.2%), other reasons were informed, and in 35 (0.1%) the selected individual was vaccinated. The non-response rate showed an increase trend throughout the study phases, from 53.8% in phase 1 to 67.9% in the last one.

## DISCUSSION

The results showed that most people in the city are still susceptible to the virus, considering the increasing number of infections during the pandemic. The prevalence of individuals with positive results was higher among black and brown people when compared with white people. Also, the prevalence was inversely associated with education level, income, and with the HDI of the primary healthcare unit coverage area (CA-PHU) of the selected dwelling. The lowest prevalences were associated with protection measures against the disease.

In 2020, several studies in Brazil and around the world aimed to estimate the prevalence of SARS-CoV-2 in their populations^15^. The results of different prevalence studies should be carefully compared, as they depend on the pandemic evolution and laboratory tests used^22^.

In our study, the estimated prevalence of antibodies against the virus in the city ranged from 9.7% (95%CI: 7.9–11.8) in phase 1 to 16% (95%CI: 13.1–19.3) in phase 10; in phase 11, when we used two laboratory tests, the estimate was 25% (95%CI: 21.7–28.7). In Brazil, Hallal et al.^20^conducted a large study involving two serological surveys in 133 “sentinel cities” in all Brazilian states. They used the same laboratory test of our study with finger prick blood samples and estimated prevalences between 0% and 25%. In the state of Espírito Santo, Gomes et al.^22^ conducted a population-based serial cross-sectional study in 11 cities and they found a prevalence of 2.1% using the Celer Technologies Inc test in finger prick blood samples. In the city of São Paulo, a study with six administrative districts found a low prevalence (4.7%) using the MAGLUMI 2019-nCoV, an *in vitro* chemiluminescence immunoassay test^21^.

In our study, the highest prevalence was initially found in the age group from 50 to 64 years, which is a vulnerable population, most affected at the beginning of the pandemic. In the following phases, the younger age groups were the most prevalent, which is the economically active population circulating around the city. This fact may be associated with the economic recover of São Paulo with the reduction of restrictions and reopening of commercial businesses^6^. Similar to other studies, there was no difference between the prevalence of men and women^16,17,20^.

Disparities regarding ethnicity and level of education are consistent with the inequalities associated with demographic, socioeconomic and risk factors for the disease transmission in the same city, as shown by Tess et al.^21^ and Rosenberg et al.^15^.

The prevalence of the virus was inversely associated with the individual’s income and with the HDI of CA-PHU – therefore, the lower the income and HDI in the CA-PHU, the higher the prevalence of SARS-CoV-2 infection. In accordance with the studies by Bermudi et al.^23^ and Menezes et al.^24^, vulnerable populations with lower income and poor housing conditions have higher risk of virus transmission and difficulties to access health services for diagnosis and treatment.

The proportion of individuals that have not reported COVID-19 symptoms since the beginning of the pandemic among those who tested positive for SARS-CoV-2 virus varied in the study phases. The proportion of up to 45.1% was high when compared with other studies. The proportion of asymptomatic individuals with 18 years or older reported in seroprevalence studies in England^18^and in a USA city (Chelsea)^25^ were 32.2% and 24.7%, respectively. In line with our study, Tess et al.^21^ found a high proportion of asymptomatic individuals living in six districts of the city of São Paulo (45.3%).

The risk associated with the work situation was lower among individuals working from home when compared to those who work at the office – this result was consistent with other seroprevalence studies of SARS-CoV-2^26,27^.

The use of masks was a protective measure against the infection and was corroborated by studies carried out in the state of Maranhão^26^ and in China^28^.

Our study has some limitations: it included a high rate of non-response, some addresses or dwellings selected in the database were not identified (29.0%), and some individuals refused to participate in the study (26.7%). In addition, venous blood samples were collected instead of finger prick to increase the sensitivity of the test, as shown by Hallal et al.^20^ and Tess et al.^21^. The large number of teams (471) composed of primary healthcare employees who were responsible for data collection may have contributed to divergences in the approach of individuals to undergo the interview process and sample collection. The design effect (deff) observed for the prevalence in the municipality and in the regions was higher than the expected for stratified samples, possibly due to the large number of strata in the study^8^. The high deff contributed to the low accuracy of the estimates; therefore, future studies should review the sample design. Individuals under 18 years of age were not included in the sample, the use of a second test in phase 11 enhanced its sensitivity, and ELISA detected 53.8% more positive cases than the rapid test. Moreover, the prevalence estimates may have been underestimated in phases 1 to 10.

Serological tests may present false-negative results in the first days of the infection; therefore, it has little diagnostic value for acute cases. The proportion of negative cases among symptomatic individuals with sample collection performed up to the 14th day of the date of symptoms onset decreased throughout the study, from 18.7% in phase 1 to 10.1% in the last phase. The probability of selecting individuals during the first 14 days of infection in the sample was decreasing and the recall bias of the date of symptoms onset was 16.4%.

In conclusion, the prevalence variation of SARS-CoV-2 infection was lower than the variation of the cumulative incidence rate, except for the last phase of the study. The differences in prevalence estimates, according to protective measures against SARS-CoV-2 infection, reinforce the need to maintain social distancing, mask use and telework in all age groups and social classes.

Sequential phases will allow the monitoring of the pandemic evolution and will verify the effectiveness of the current recommended protection measures for the population. Further studies should consider vaccinated people and the influence of vaccination on the population.
